# Building multi-system nexuses in low-carbon transitions: Conflicts and asymmetric adjustments in Norwegian ferry electrification

**DOI:** 10.1073/pnas.2207746120

**Published:** 2023-11-13

**Authors:** Allan Dahl Andersen, Frank W. Geels, Markus Steen, Markus M. Bugge

**Affiliations:** ^a^Centre for Technology, Innovation and Culture, University of Oslo, 0851 Oslo, Norway; ^b^Department of Food and Resource Economics, University of Copenhagen, 1958 Frederiksberg C, Denmark; ^c^Division of Innovation, Management and Policy, Manchester Institute of Innovation Research, The University of Manchester, Manchester M15 6PB, United Kingdom; ^d^Division of Technology Management, SINTEF Digital, 7465 Trondheim, Norway; ^e^Nordic Institute for Studies in Innovation, Research and Education, 0653 Oslo, Norway

**Keywords:** Nexus building, multi-system interaction, low-carbon transition, electrification, maritime transport

## Abstract

While nexus research in sustainability science has investigated the consequences of connected systems, it has paid less attention to the processes of building nexuses which is becoming increasingly important in low-carbon transitions because these often require the creation of new connections between multiple consumption–production systems. Building on multi-system research in the sustainability transitions literature, this paper introduces a conceptual system interface perspective on nexus-building which considers four dimensions (technology, actors, institutions, and resources) that are useful for analyzing nexus-building dynamics. We apply our framework to the case of electrification of ferries in Norway which requires the building of a new interface between the electricity system and the maritime transport system. The case study shows that the system interface was initially characterized by conflicts and tensions in all dimensions, which actors then attempted to resolve through cross-system intermediation and adjustment activities. These activities were asymmetrical because of differences in external pressures, urgency, unequal power relationships, and different degrees of interest in cross-system nexus building. Because important tensions remained unresolved, ferry actors started implementing sub-optimal workaround solutions in the diffusion phase.

## Significance

Achieving urgent low-carbon transitions requires not only the rapid deployment of low-carbon innovations (such as electric vehicles, heat pumps, electric arc furnaces, electric boilers, and bio-based products) in particular systems, but also the building of connections to other systems to support these innovations through resource flows. Our research shows that building connections across systems is characterized by conflicts and tensions because actors in both systems have different interests and preferences for technical solutions and regulations. Because various actors often initially underestimate these challenges, conflicts and tensions often only appear in full in later transition phases. Since multi-system nexus building is already beginning to cause delays in low-carbon transitions, policymakers should dedicate more attention and resources to the issue.

Climate change mitigation will require low-carbon transitions across all consumption–production systems including mobility, heating, buildings, electricity, agri-food, and the production and use of basic materials such as steel, chemicals, and cement (1). Most research in transition studies has understandably focused on drivers, barriers, and policies in single systems, where transitions urgently need to accelerate (2). However, since no system functions in isolation, most transitions will require inputs from or have effects on other consumption–production systems. Multi-system interactions in transitions are thus likely to become a more important topic for research and policymakers in the coming years.

Nexus research in sustainability science has long recognized that systems interact with each other, with many scholars focusing on sector couplings (3, 4) and the nexus between water, energy, and food (5, 6). Frequent issues of concern are competition for natural resources between systems (e.g., using water for hydropower or irrigation) and unintended consequences and system spillovers (e.g., biofuel production causing water scarcities and food price rises), leading to calls for more policy coherence (e.g., integrated water management, integrated natural resources management) and more integrated research (e.g., integrated assessment modelling of biophysical resource flows).

Despite the rapid increase of nexus research, sustainability science scholars have noted that the nexus is “not a clearly defined construct or an agreed and tested framework” (5) and that “understandings and usage of the term nexus are plural, fragmented, and ambiguous” (7). Indeed, researchers are yet to develop a generic conceptualization of nexuses, reflecting that “application and implementation of nexus approaches are still in their infancy” (6). For this reason, Clark and Harley (8) see a need for disentangling in more detail what actually happens in multi-system nexuses, while Liu et al. (6) call for more research “exploring mechanisms underlying nexus dynamics”. Moreover, nexus research has so far focused more on the consequences of connections between systems (such as spillovers and resource competition) than on the connections themselves. But if sustainability science wants to develop further towards solutions-oriented research (9), a more explicit and processual understanding of nexus dynamics would arguably be helpful. Especially the building of new nexuses between systems is an important but understudied topic both in sustainability science and transition studies.

To move the nexus discussion from a focus on consequences towards a focus on nexus dynamics and solutions, we address the following research question: What are the main processes in nexus building between multiple systems in low-carbon transitions?

To answer that question, the paper develops a system interface perspective on multi-system nexuses, which utilizes the analytical elements (actors, institutions, resources, technologies) identified by Clark and Harley’s seminal (8) overview of sustainability science, but mobilizes them somewhat differently because of our focus on transitions and nexus building. In particular, we will use approaches to multi-system interactions in transition studies based on the multilevel perspective to make a first step towards conceptualizing the dynamics of the “between-systems” interface domain including core processes in multi-system nexus building. With this perspective we conceptualize and define the nexus itself as a multi-system interface where actors from different systems meet, discuss, and negotiate actor roles, institutions, technical designs, and standards that in combination set the conditions for cross-system material resource flows. Because different systems have developed independently and are stabilized by lock-in mechanisms across technology, resource, actor, and institutional dimensions (10, 11), it is unlikely that actors from different systems are equally keen to develop multi-system nexuses and make significant adjustments or investments. Our perspective therefore suggests that nexus building is initially characterized by tensions and conflicts, which is subsequently followed by streamlining efforts, which may be asymmetric between actors and involve workaround solutions if conflicts cannot be resolved through negotiations.

We will evaluate and explore the conceptual framework with an illustrative case study of the electrification of the maritime transport system in Norway focusing on passenger ferries. The transition from diesel to electric ferries also involved the building of new nexuses with the electricity system to enable the charging of electric ferries. While this new nexus may, at first glance, appear beneficial for both systems, our analysis will show multiple cross-system tensions and conflicts.

The paper is structured as follows. Section 1 describes our system interface perspective, which mobilizes insights from sustainability transition studies. Section 2 discusses research methods and delineates the case. Section 3 presents our case study. Section 4 discusses the findings and section 5 concludes.

## 1. System Interface Perspective

**Fig. 1.**
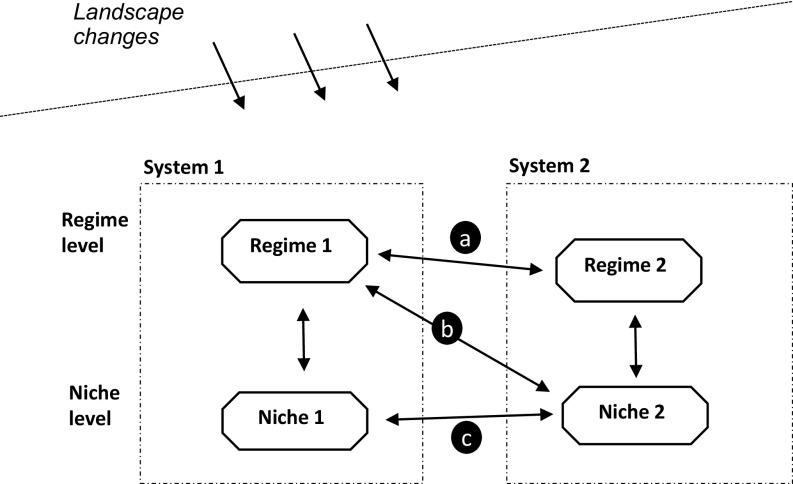
Three patterns in multi-system interactions (building on refs. 13 and 16).

Sustainability transitions research investigates the dynamics of fundamental changes in consumption–production systems aimed at addressing persistent socioenvironmental problems (12). While most transitions research has focused on single systems, there is an emerging research stream on multi-system interactions in sustainability transitions (13–16). Drawing on the multilevel perspective (17, 18), which conceptualizes transitions as multidimensional struggles between radical niche innovations and existing regimes in a focal production–consumption system in the context of slow-changing “landscape” developments, this research stream typically analyzes multi-system dynamics as a) regime–regime, b) regime–niche, or c) niche–niche interactions (Fig. 1). Scholars also identified different types of interaction such as competition, symbiosis^*^, integration, and spillover (15) and distinguished functional couplings (interdependency due to resource exchanges) and structural couplings between core elements of consumption–production (or “sociotechnical”) systems such as actors, technologies, and institutions (14).

While such abstract, system-level typologies are helpful for a general understanding, they say less about the more specific microlevel dynamics of multi-system interactions that underpin the arrows in Fig. 1. Rosenbloom (13) therefore suggested that future research should develop a more granular and processual understanding of how such interactions happen in practice.

We aim to further develop this suggestion by investigating processes of nexus building as the creation of new multi-system couplings. We conceptualize a nexus as a point of interconnection between two consumption–production systems and suggest that the low-carbon electrification of systems like mobility, heating, and manufacturing will require the building of new interfaces (or nexuses) with the electricity system to establish new electricity flows.

Drawing on sustainability science (8) and sociotechnical transitions research (17, 18), we suggest that nexus-building processes have technological, agentic, institutional, and material resource dimensions. Our conceptual framework will discuss core processes for each dimension. Whereas the bidirectional arrows in Fig. 1 implicitly assume that nexus building is a collaborative endeavor, our framework assumes that different systems have evolved independently and that actors in both systems may not be equally interested in nexus building. This means that early phases of nexus building are likely characterized by tensions and conflicts and that later phases show more efforts at streamlining and adjustments, which may be asymmetrical between actors. While some of our core processes (like learning processes, experimentation, network building, and institutional adjustments) are familiar to transition scholars (19, 20), an important novel aspect in our framework is the role of conflict, tensions, and asymmetry in their instantiation in multi-system nexus building.

Transitions research (17, 18) typically distinguishes four phases in transitions: 1) experimentation in protected pilot or demonstration projects; 2) stabilization and early deployment in market niches; 3) mass diffusion into mainstream markets; and 4) anchoring and reconfiguration of a wider system. Applying this perspective to interface dynamics, we expect conflicts and tensions to be limited in the first phase because lead actors sign up voluntarily to pilot projects which tend to have dedicated nexus arrangements (e.g., regulatory exemptions or carried out in ideal location). We expect more conflicts and tensions in the second phase, when deployment of a niche innovation (like electric ferries in the maritime system) also requires wider nexus building with another system (like electricity) and involvement of mainstream actors who are less interested in nexus building. Wider diffusion in the third phase will likely involve efforts to reduce conflicts and tensions in the interface, which may involve negotiation and adjustment, or workaround solutions to circumvent continuing tensions. In the fourth phase, we expect a stabilization of rules, roles, and technical issues in the nexus such that resource flows happen via standardized transactions. We now further discuss core tensions and processes for each dimension in the second and third transition phases.

The technological dimension of nexus building is about achieving technical compatibility between two different systems, which typically requires the construction of a physical link between two systems (such as cables or pipes) to enable material resource flows, and may additionally involve “interface technologies” to generate technical compatibility among otherwise incompatible modules or subsystems in a wider system (21). Electric ferries, for example, require not just cables to connect to the electricity distribution grid but also charging technologies to charge ferry batteries. These interface technologies, in particular, may initially exist as a variety of competing design options with different technical, economic, and operational characteristics that may imply different responsibilities and costs for various actors (22). This technical variation and uncertainty may be a source of tension and conflict in nexus building if actors from different systems disagree about preferred options. While the transitions literature often assumes that such conflicts in early transition phases can be overcome through learning processes, which pave the way for wider diffusion (19, 20), we suggest that this may not be the case for nexus building because of diverging interests or power asymmetries between resourceful actors from different systems. If that is the case, then actors from one system may be forced to implement suboptimal or workaround technical solutions to create nexuses that support the diffusing niche innovation.

The institutional dimension of system interfaces refers to the need for achieving compatibility between the diverse institutional arrangements in interacting systems by establishing a set of rules that allow actors to build and operate a technological interface. The problem in early phases of nexus building is that the formal institutions (e.g., laws regulations, policies) and informal institutions (e.g., norms, shared beliefs) (23) in different systems are likely to misalign, guiding actors in different directions such that they are disincentivized to collaborate or disinclined to work on technological system interfaces. Diverging institutions can thus initially lead to conflicts, tensions, and bottlenecks in nexus building (13, 24).

To enable wider diffusion of niche innovations in later phases, actors will subsequently make institutional adjustments, which may involve negotiation and communication between different perspectives (24, 25). While transition scholars have previously studied these kinds of processes between emerging niche- innovations and existing regimes, nexus building implies interactions between multiple incumbent actors and locked-in regimes, which means that institutional adjustments may be more difficult and conflictual. If there are power asymmetries between the two systems, it is possible that actors in one system will be forced to make most institutional adjustments.

Nexus building also involves interactions between actors from different systems to create technological connections, adjust institutions, or develop new roles and work arrangements. These cross-system interactions may initially be challenging because actors are embedded in system-specific organizational fields (26, 27), which means they are initially more oriented towards other actors in “their” system than towards actors from other systems. Early cross-system interactions may therefore be characterized by reticence and misunderstandings. When actors have different interests, competencies, or preferences, there may additionally be conflicts and disagreements in the system interface.^†^

To facilitate broader diffusion of niche -innovations, actors may create new cross-system meeting places or networks to discuss and negotiate issues, exchange information, or work towards standardized solutions (28). Actors may also engage in learning processes to articulate new roles, relationships, and institutions (29) or acquire new competencies that enable substantive collaboration (30). They can also hire new people and change organizational structures. Actor engagement in these processes may be asymmetrical due to differences in power between systems and possibly insufficient to resolve all conflicts.

Material resources are another important dimension because a prime goal of nexus building is the creation of connections that enable resource flows between systems. Transition studies have, however, paid less attention to resource flows than sustainability science (5, 6), which is something this paper can only begin to remedy. Drawing on sustainability science (8), which sees consumption–production systems as embedded in a biophysical environment with which it exchanges resources, we suggest that resource flows are also important in multi-system interactions. We take a broad view on material resources, which can include biophysical entities and technical components as well as fuels and electricity.

The abundance or scarcity of a particular material resource has historically shaped the direction of technological innovation and motivated actors to build new nexuses across systems (31), e.g., oil for mid-twentieth century transitions in transport, agriculture, and chemicals (32), or the role of wood scarcity in the UK steamship transition (17). If resource flows between systems require new or adjusted technological connections, this may cause early disagreements about who pays and who benefits, because such changes are often costly (33). In later transition phases, conflicts may be more about resource competition, as sustainability science scholars suggest (6). The emergence of electrification as climate mitigation meta-strategy (34), for instance, may imply that multiple systems compete for connections to the electricity grid.

**Fig. 2.**
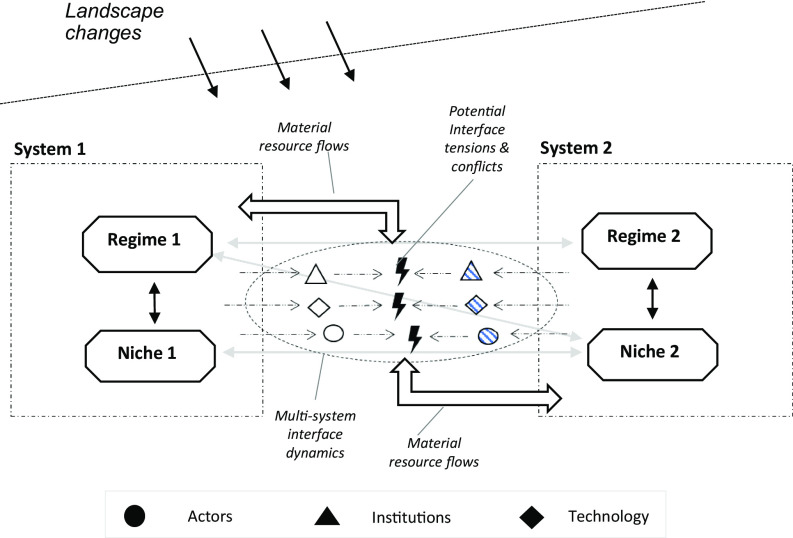
A granular, microlevel perspective on the multi-system interface.

Our granular, microlevel perspective, which is schematically visualized in Fig. 2, draws attention to several dimensions of multi-system interaction and to possible tensions and conflicts. The perspective also indicates core processes in each dimension and how these may differ across transition phases. We will further explore our system interface perspective, which builds on and reorients existing transition studies’ concepts, with a case study of electrification of the Norwegian maritime system.

## 2. Research Design

We use a single case study research design, which is well suited for exploring new phenomena like nexus building in low-carbon transitions and new approaches like our interface perspective (35). We selected the case of ferry electrification in Norway because it includes the building of a new system interface between the maritime system and electricity system, especially the grid. It is thus a relevant case for gaining new analytical insights about nexus building in low-carbon transitions. In terms of Fig. 1, the case qualifies as cross-system interactions between a niche innovation (electric ferries) in the maritime system and an existing regime in the electricity system. At an abstract system level, these interactions can be qualified as symbiotic because ferries benefit from low-carbon electricity (resources), while grid actors benefit by selling more electricity. Our more detailed microlevel analysis of various processes will, however, show many conflicts, disagreements, power struggles, and asymmetric adjustments to establish the nexus. The case is also interesting because Norway is a global frontrunner in low-carbon electrification in general and in the maritime system in particular. It can thus reveal insights about nexus-building processes that may be relevant in other contexts.

**Fig. 3.**
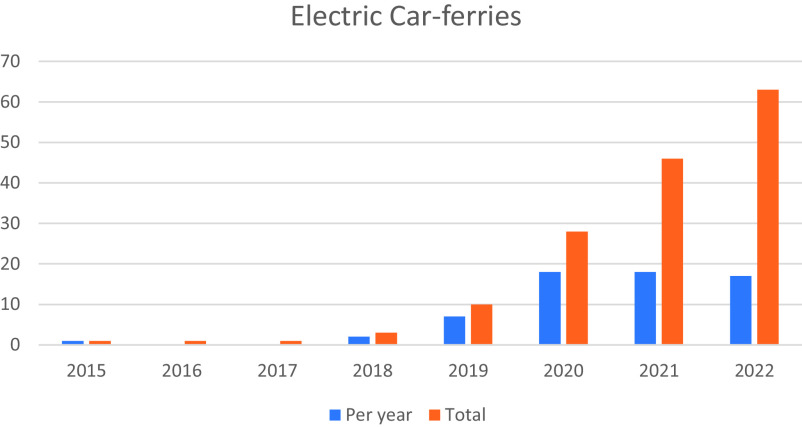
Number of electric car ferries in Norway, 2015 to 2022 (36).

In terms of temporal demarcation, we focus on the early deployment period (2015 to 2018), when preparations for widespread use of electric ferries brought tensions and conflicts in the system interface to the fore, and the subsequent diffusion period (2018 to 2022), when actors tried to address various tensions and conflicts through adjustments and negotiations. By late 2022, there were 63 electric ferries operating on 44 of Norway’s 133 near-shore car ferry routes, see Fig. 3. Because Norway has many fjords, these car ferries are integral to functioning of the road network, transferring vehicles and people along the coast.

Central actors in the maritime system are the formal owners of Norway’s 133 car passenger ferry routes including county municipalities (county roads) and the Norwegian Public Roads Administration (NPRA) (national roads). These route owners contract ferry operators for providing transport services via public procurement. The route owners set the terms for contracts, which typically last for 10 y. Within a new contract, operators sometimes contract technology suppliers to construct new ferries or retrofit existing ones (D8). Central actors in the electricity distribution system include the 124 regional grid companies (Distribution System Operators, DSOs) and the national grid company (Transmission System Operator). The national regulator, NVE (Norges vassdrags- og energidirektorat) oversees and enforces the regulatory institutions that aim to promote efficient power sales, cost-efficient energy systems, and efficient energy use (37).

The case study draws on multiple data sources. An important source of primary data is interviews with 28 respondents (*SI Appendix*), who we selected strategically based on two criteria. First, selected actors must have experience in the emerging interface because that is our phenomenon of interest. Second, our population of selected actors should cover all key actor types, discussed above. We additionally interviewed actors who were involved in both systems, namely DNV (a consultancy company), ENOVA (an innovation funding agency), and the Zero Emission Resource Organization (an environmental NGO). We selected interviewees in high-level management positions who are knowledgeable about their organization and its environment. Interviews were conducted via video call, lasting between 1 and 2 h. The interview guide focused on drivers and barriers for ferry electrification with particular attention to interactions across systems in terms of actors, institutions, technology, and resources. Interviews were conducted in 2018 and early 2019, when the transition was shifting between the two phases which was suitable for understanding conflicts, tensions, and challenges in the early period as well as initial responses and solutions in the later phase. In addition to the authors’ deep knowledge of the case history, other important data sources include government documents, industry reports, media articles, scientific literature, and participation in industry seminars (see overview in *SI Appendix*). We triangulated the multiple data sources in our illustrative and selective case study, which aims to explore and test our conceptual framework.

All interviews were recorded, transcribed, and subsequently coded. The process started by developing a coding scheme based on the conceptual framework. Several iterative test rounds of coding were then conducted to align the coding scheme, other data sources, and empirical observations. We looked for and coded events and activities involving tensions in the system interface and actor responses to them (e.g., cross-system intermediation work). In the third step, we synthesized results of coding and looked for recurring patterns. We reference our empirical data, which is listed in *SI Appendix*, with assigned codes in brackets including interviews (R1, R2) and key documents (D1, D2). The case study describes selected developments covering the four analytical dimensions of our framework.

## 3. Case Study

The political context of the case is that the Norwegian government in the early 2010s introduced ambitious climate targets including reducing greenhouse gases (GHG) emissions by at least 40% by 2030. Because Norway already had a low-carbon electricity system, where renewables account for approximately 98% of electricity generation (38), policymakers targeted GHG emission reductions in other systems such as transport and industry and typically via low-carbon electrification.

In this context, the NPRA in 2011 introduced a new public procurement innovation tender to reduce ferry emissions containing both higher demands for environmental performance and financial support for building a low-carbon ferry (R18). It was won by ferry operator Norled and grid company BKK Grid, who subsequently led the Ampere demonstration project (2011 to 2014) with one electric ferry across the Sognefjord. They were exempted from complying with all formal regulations and therefore did not experience institutional tensions in the project. They also did not experience technical interface challenges because the project used a stationary battery system for electric charging rather than grid capacity expansion (R10, R13). Interface challenges therefore did not appear until electric ferries started to be deployed outside the protective niche.

The success of the Ampere project paved the way for a parliamentary decision in 2015 that demanded that all ferries in Norway would be zero-emission by 2025. This led the NPRA and counties to start implementing zero-emission requirements more widely in their ferry contract tenders (39). Ferry operators responded by preparing plans, project proposals, and technologies for electric ferry implementation.

### 3.1. System Interface Tensions in Early Deployment (2015 to 2018).

#### Material resource flows.

Despite abundant electricity production, ferry electrification experienced a shortage of electricity flows, i.e., transporting electricity in the right amount at the right time to the right place. Ferry electrification is challenging because ferry quays are often in remote areas with weak grids, which struggle to provide the relatively large electricity flows required for charging the batteries of electric ferries (on average 2 to 4 MW but sometimes up to 10 MW over a period of 5 to 15 min).

#### Technology.

Ferry operators initially focused on fast charging directly from the grid while the ferry was docked. DSOs were often more reluctant, however, because it would require them to upgrade the local grid, which is demanding, expensive, and can take years. To avoid this, DSOs would often recommend that ferry operators install stationary onshore battery systems that could recharge slowly from the grid but discharge rapidly once the ferry arrives. But ferry operators were reluctant to manage such onshore battery charging systems leading to tensions (see below).

In this phase, ferry operators experienced challenges related to technological variety in the charging interface. Because vessels and grid conditions varied between locations, and because public procurement contracts varied between ferry routes, ferry operators initially developed site-specific solutions for charging technologies and vessels in collaboration with technology suppliers. This resulted in technical variation in automated docking (e.g., mechanical, vacuum, and magnetic options), charging connections (different locations on vessel, different types of plugs and pantographs), and charging technologies (e.g., direct fast charging or stationary battery systems) (R13), see illustration in *SI Appendix*. This lack of standardization created uncertainties for ferry operators who had to invest time and effort in working out site-specific solutions once they had won a contract. This left them and their suppliers only about 3 years for designing, ordering, and building electric ferries, charging configurations, and grid connections, which was demanding as one ferry operator said: “We had technology development and production almost in parallel because there were very clear deadlines for when it should be ready. This was probably one of the biggest challenges” (R5).

The lack of standardization created challenges for NPRA, who needed to approve charging technology choices at each quay but lacked in-depth technical competence of different configurations creating tensions and bottlenecks in the process. One ferry operator therefore complained that: “they assess lots of different technology designs…we have not made final decisions on charging technology because we are waiting for a response from NPRA” (R13).

The pace of change and lack of standardization moreover precluded real-world testing of all technologies, which meant that some problems only appeared when deployment started, including impact on wider grid, and cable types and sizes (R16). One DSO retrospectively observed that “We should have had more full-scale demonstration projects to prepare properly. Instead we went from a small demonstration project (Ampere) directly to mass diffusion” (R10).

#### Formal and informal institutions.

The DSO’s aversion to direct fast charging and grid upgrades partly related to their formal revenue model. As a regulated natural monopoly, DSOs receive yearly income from the regulator based on a model that distributes revenue based on relative productivity between DSOs. To optimize the use of existing assets and reduce costs for taxpayers, the model incentivizes DSOs to maximize the number of customers (sales points) served and minimize capital cost. This disincentivized DSOs to engage with ferry electrification because it would require them to make many (expensive) changes without notable income increases, as a DSO interviewee explained: “The revenue model has worked well historically for running a cost-efficient system, but it doesn’t fit for electric ferries where you need major investment in peripheral parts of the grid” (R11). Furthermore, high electricity flows during ferry charging would increase electricity losses and increase “wear and tear” on grid equipment, which thus generates costs that the revenue model does not compensate DSOs for (R10). This led to conflicts with ferry operators that did not understand these operational concerns of DSOs and instead perceived them as uninterested and nonsupportive (R5).

When the NPRA introduced their new ferry route contracts, their ferry division paid little attention to the new system interface, i.e., electricity provision (R18). They spoke with ferry operators about these new procurement contracts but largely ignored electric utilities and DSOs. This neglect was not deliberate but resulted from organizational norms and mindsets focusing on issues at sea, which more generally led maritime transportation actors to ignore what takes places onshore. An NPRA interviewee noted that: “Traditionally, the ferry and the quay have been [institutionally and organizationally] separated. This is a precondition for the transportation service we procure. But that interface has become much more complicated as a consequence of the green transformation” (R18).

The lack of attention to the electricity system led to situations where ferry operators prepared their contract bid assuming that electric charging would be provided by expanded grids. They often made this assumption without properly consulting the local DSO, who would sometimes recommend another solution. The initial lack of early consultation was further complicated by regulatory institutions in electricity, as an environmental NGO explained: “Only when ferry operators have won a contract can they order the electricity they need from the DSO, and by then it is too late” (R24).

Institutional challenges related to different assumptions about time frames in both systems also appeared. Because DSOs traditionally focused on optimizing existing assets rather than on engaging with innovation, they assessed the need for new capital expenditures only after they received new grid connection requests, which would then be analyzed on economic cost-efficiency ground. DSOs must also often hold public consultations (or start land expropriation procedures) before they can construct new grids, which typically takes years (R10). Electricity system actors thus work in multiyear time frames and follow rigorous procedures. The electricity system regulator explained: “It is the same [rules] for everyone regardless of whether the activity of the user is considered societally worthless or very valuable” (R15). DSOs thus experienced that ferry operators expected grid transformation to happen much faster than was normal or possible in their view.

These differences created conflicts across systems. Ferry operators were indeed impatient and were frustrated by the DSOs’ slow grid connection building and lack of proactive engagement. One interviewee commented that “Currently, DSOs do not do anything before they get a formal request, and then, they are typically surprised by how much electricity ferry operators want” (R24). The Zero Emission Resource Organization and the DSO business association also criticized the electricity regulator NVE for being too slow in changing incentives for DSOs: “NVE should be more proactive and anticipatory towards the challenges that could emerge from wide electrification” (R16).

#### Actors and coordination.

As implementation of electric ferries progressed, ferry operators had to engage more with grid access and charging technology. They therefore articulated new roles and responsibilities, which deviated from their traditional sea-focused roles. Ferry operators initially felt uneasy about these new roles because they lacked electricity-related competencies and thus perceived the charging interface as risky territory (R13, R22). Ferry operators generally thought that DSOs should own and operate battery packs as extensions of the grid while the procuring actor (NPRA or counties) should own the charging technology, leaving ferry operators to focus on ferry operations (R19). Ferry operators also had contractual reasons to avoid onshore investments, because the lifetime of the assets is 30 to 40 y while the ferry operator contract is for 10 y (R18). DSOs, however, were not interested in operating battery equipment beyond the distribution grid, because such batteries cannot be used “cost-efficiently” under current regulations (e.g., providing flexibility services in the grid) (R10, R15). These tensions and conflicts about roles and responsibilities led to variation, with some ferry operators reluctantly installing battery pack charging options to avoid long grid connection waiting times, while others refused that option, causing delays in electric ferry operation.

Early nexus building was also hampered by various cross-system coordination tensions. Ferry operators and DSOs, for instance, complained that their interactions with NPRA and county municipalities were hampered by fragmentation and unevenness of technical and contractual competencies. One DSO noted that: “There is not a strong actor which can both think rationally in terms of standardization and that can build sufficient knowledge in the area… this is problematic when you have very complex economic and technical challenges to deal with” (R11).

There was also limited coordination and knowledge exchange between the 124 DSOs (R22). This led to DSOs in different parts of the country having diverging views on technical specifications, timelines, and feasibility (R3, R5, and R16), which created challenges for larger ferry operators with multiple ferry routes who had to negotiate in parallel with multiple DSOs with different demands.

Cross-system efforts to share experiences between nexus-building projects or stimulate discussions between wider actor communities to standardize and aggregate lessons were negligible (R8). The DSO business association noted that: “The main mistake [in electrification planning] was that it took too long before actors [from different systems] started to collaborate” (R16).

This limited cross-system coordination meant that early nexus building happened on a project-by-project basis, leading to high diversity and ad hoc solutions without standardized procedures, which made the entire process of ferry electrification more cumbersome, costly, and time-consuming (R15, R16). It also led to lasting conflicts within projects, because of the monopoly power position of DSOs, as the regulator noted: “Negotiations between a customer and a monopolist is not equal at all” (R15).

### 3.2. Adjustments and Workaround Solutions during Diffusion (2018 to 2022).

In the diffusion period, actors made various adjustments to alleviate system interface conflicts and tensions. These adjustments were however asymmetric between the systems and did not fully resolve all problems.

#### Material resource flows.

Grid-related challenges increased in this period as electric ferry diffusion led to more grid connection requests. In addition, competition for electricity flows increased as electrification became an important decarbonization strategy for multiple systems (e.g., transport, industry, buildings, construction). These other systems experienced similar challenges as electric ferries, which led to growing complaints about slow grid connections (40). In 2021, the Norwegian government therefore launched a “Grid development commission” to investigate the problems and provide recommendations.

#### Technology.

To reduce technological uncertainties and simplify implementation, there were streamlining efforts. In 2018, the business association for DSOs (REN) started to develop a technical guide on ferry charging and a template for negotiation and communication between DSOs and ferry operators, but this mainly aimed at improving the maritime actors’ understanding of how the electricity system works. The Norwegian Electro-technical Committee also started creating new-to-the-world technical standards for how to connect ferries to the grid, but this proceeded slowly because of ambitions to implement these in the European Committee for Electrotechnical Standardization (R16).

Nevertheless, variety in charging technology and on-vessel battery systems continued to exist during the diffusion process, because technological immaturity and the diversity of ferry routes precluded making definitive choices about standards for the best technical configuration (41). While ferry actors were increasingly operating electric ferries (Fig. 3), they were still tinkering and experimenting with charging technologies including docking processes, communication between on- and offshore systems, and figuring out where plug and charger should be located on ship and quay, respectively. Because DSOs continued to be slow with grid connections and upgrades, ferry operators continued to work on workaround solutions such as stationary battery pack systems, which were complemented in this period with experiments with floating, stationary battery system (when space on the quay was limited) and experiments using onshore battery packs to provide electric vehicle charging and flexibility services to the grid. Since 2020, they also started experimenting with battery-swapping, which reduces the need for new grids (42, 43). The lack of technical interface standardization and continued uncertainties caused delays in ferry electrification, with one ferry operator stating in 2020 that “the ship-to-shore [i.e., charging] is the weakest link in the chain for electric ferries” (44).

#### Formal and informal institutions.

In response to criticisms and continued problems, the electricity system regulator initiated a review process in 2018 of the DSO revenue model. This was supposed to last 2 y but is still ongoing at the time of writing. The regulator therefore has not yet adjusted the financial incentive regulations for DSOs, which continues to hamper DSO engagement with ferry charging (45, D12). Instead of changing regulations, the regulator (NVE) has dedicated some efforts to clarifying different aspects of regulation to improve the maritime actors’ understanding of the electricity system (R15).

Maritime actors made more institutional adjustments to alleviate charging and grid connection problems. In 2018, the NPRA changed its procurement procedures for new ferry routes, aimed at engaging more with DSOs in the preparation of tenders. The NPRA would, for instance, ask DSOs how much capacity they had available and what the costs were for different degrees of grid upgrading. The NPRA would then include this information in new procurement specifications, expecting ferry operators to address this in their proposals. The NPRA also lengthened procurement procedures from three to four years before electric vessels are meant to be operative, which gave ferry operators more time to prepare grid connections. These adjustments reduced the risk of conflictual stalemates between DSOs and ferry operators and helped accelerate electrification projects.

In some places, ferry operators and the NPRA also changed ferry timetables to better align with grid charging capacity. By sailing slower and extending charging time at quay, vessels needed less power and less grid capacity when charging. On routes with multiple vessels, timetable adjustments ensured that they do not dock and charge at the same time. Timetable adjustments thus reduced the need for grid upgrades in some places (R21).

#### Actors and coordination.

Maritime actors engaged in substantial learning and competence building this period. All ferry operators developed new competencies on electric systems, electric motors, batteries, and charging to be able to apply for tenders. The NPRA hired technical consultants, expanded their knowledge about the electricity system and reorganized to create new divisions with responsibilities for sea, onshore equipment, and the emerging electrocompetence (R18). Several county municipalities also developed new competencies on electric ferries and charging (R11, R22), which was supported by the NPRA and by new system interface actors (R5, R18).

Electricity system actors did not dedicate much effort to learning about ferries and maritime actors. DSOs did not prioritize ferry operators, which they perceived as just one actor group among many they must engage with. As electrification has become a more widespread decarbonization strategy, DSOs have been overwhelmed by demands from many systems, which often have intermittent electricity and grid demands and higher maximum usage than “normal” electricity consumers (D12). In 2019, the business association for the electricity system, Energi Norge, developed guidelines for how new large grid customers should engage with DSOs and what they can expect (46), but this focused more on one-directional information provision rather than mutual learning. Except for some technical and communication guidelines, and participation in electrification conferences, DSOs did not work to improve coordination with ferry actors.

Cross-system coordination improved somewhat in this period because the NPRA developed new interaction modes with DSOs and because electricity system actors developed technical and communication guidelines. These initiatives were, however, mainly aimed at improving generic procedures between system-level actors. Cross-system coordination between operational actors such as DSOs, ferry operators, and technology providers has remained ad hoc. The Zero Emission Resource Organization (environmental NGO) also tried to improve cross-system intermediation by publishing reports, writing media news items, and organizing a cross-system conference in 2018 focused on the system interface (i.e., charging) with participating actors from maritime and electricity systems. This stimulated mutual understanding and coordination across both systems but did not resolve structural misalignment problems.

Moreover, because ferry operators, DSOs, and municipalities remained reluctant to articulate new roles and responsibilities in the charging technology space, new hybrid actors emerged to fill this void by developing new business models. The new “electrification company” Eviny Elektrifisering (created by an electric utility) and PLUG AS (a joint venture between a port company and an electric utility), for example, started providing consultancy services, while also offering to own and operate the charging technology and system interface (R2). These new hybrid actors thus offered to “take care of the middle part…and tidy up the garden” (R3) and started to explore other opportunities such as using ferry charging points to provide charging services to other vessels, cars, and trucks and provide balancing services to the grid (47). By taking up system interface roles, these new actors helped to mitigate charging challenges without existing actors having to substantially change their roles.

## 4. Discussion

The case study clearly showed that nexus building is challenging, even in transitions with symbiotic multi-system interactions, where nexus building seems relatively easy and beneficial for both systems. The case study also showed that our conceptual system interface perspective is useful for analyzing nexus-building processes across four dimensions (technologies, resources, institutions, and actors). We found that nexus building in the early deployment phase of electric ferries (2015 to 2018) was hampered by conflicts, tensions, and misalignments across institutions (e.g., disincentivizing revenue models, myopic procurement practice, and different operational timeframes), technologies (e.g., diversity of interface technologies, lacking standardization, uncertainties, diverging preferences), and actors (e.g., lacking knowledge of other system, disagreement over actor roles, absence of cross-system forums or platforms where actors could negotiate, share experiences, and establish productive working relationships), which led to challenges in providing the required resource flows.

The diffusion phase of electric ferries (2018 to 2022) was characterized by multidimensional streamlining and adjustment processes to address the nexus-building challenges, including the development of technical and communication guidelines, amended ferry timetables, and ferry route procurement procedures, electricity-related competence building by maritime actors, the creation of cross-system platforms, forums, and conferences, and the emergence of new interface actors providing charging services. An important finding, which confirms our conceptual arguments in section 1, is that these system interface adjustments were asymmetrical, with maritime actors making significantly more amendments than electricity system actors. One reason for this asymmetry is that the nexus building demand primarily came from the maritime system, where actors are under pressure to achieve a politically imposed decarbonization goal that drove ferry electrification. The electricity distribution system actors did not face a similar pressure to build nexuses with the ferry system. In fact, they increasingly face grid connection demands from many other systems, which means that they see ferry operators as only one among many new customers. Another reason for the asymmetry is that DSOs have more power because they are monopoly actors, who primarily work under the obligation to operate the grid cost-efficiently. Because of these different pressures, maritime actors were forced to undertake more innovation, adaptation, and intermediation activities to succeed with electrification than the electricity system actors.

Another important finding is that several system-interface conflicts, tensions, and misalignments were not fully resolved in the diffusion phase. Indeed, multiple nexus-building issues (e.g., charging technology variety, DSO revenue model, mutual understandings, collaborative working arrangements, effective cross-system coordination) remained problematic and insufficiently addressed, which continued to cause problems and delays for electric ferries. Nexus-building process for ferry electrification thus has not yet stabilized and is still unfolding, requiring (mostly maritime) actors to improvise and address problems on the go, which in several instances led to various workaround solutions.

This finding is conceptually interesting because it deviates from existing transitions theory, which suggests that alignment challenges are resolved in the experimentation and early deployment phases so that the innovation and its wider system stabilize before mass diffusion (48). One reason for the unresolved nexus problems is the asymmetric actor engagement we discussed above. While maritime transport actors were in a hurry to meet politically imposed decarbonization targets, electricity system actors operated within an a-temporal optimization orientation, where it is more important that things happen efficiently than quickly (49).

Another reason is that policymakers cut short the emergence phase and the possibilities for more extensive learning-by-doing through experiments and pilot projects. Although transitions theory (19, 20) typically recommends sequences of multiple pilot projects, there was only one pilot project (Ampere) in the electric ferry case, after which policy changes in 2015 led to rapid deployment across nearly all routes. This temporal compression limited the available time for trial-and-error learning processes in the experimentation phase and required ferry operators to include immature and uncertain technologies in their bids, which de facto merged competitive procurement procedures and innovation processes. This created high risk for ferry operators and led to some proposals that turned out to be based on wrong assumptions about the charging interface. In some instances, slow grid expansion forced ferry operators unwillingly to invest in onshore battery system to meet project delivery times or implement other workaround solutions.

**Fig. 4.**
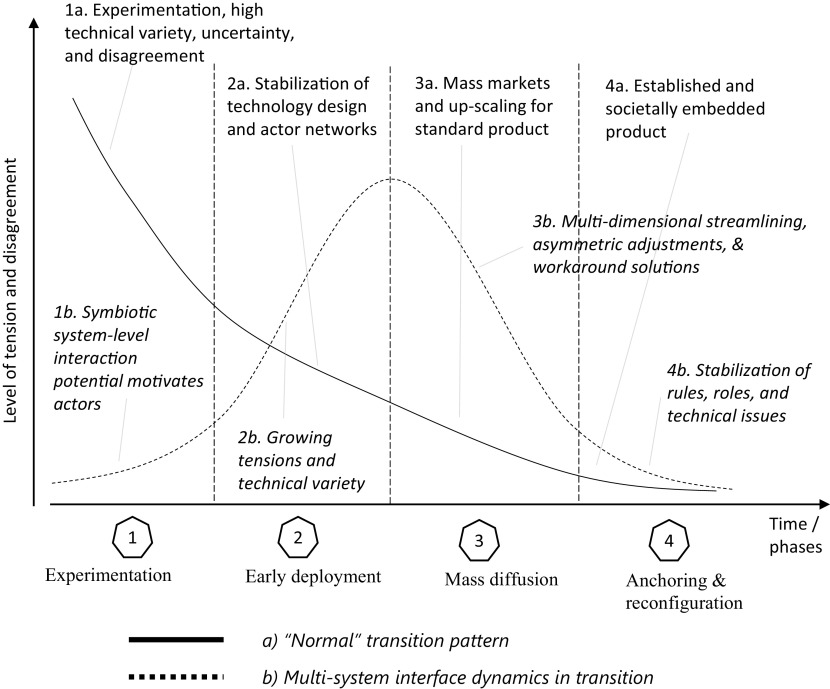
Level of tension about design specificities across transition phases in normal transitions and in multi-system interface dynamics during transitions.

With regard to technological designs, our study thus shows that the dynamics of tensions of emerging innovations differ between “normal transitions”, where technological variety and disagreement about possible design options is initially high but then reduced through learning processes and social interactions (19, 20), and “multi-system interface dynamics”, where tensions and disagreements over technical designs are initially low but subsequently grow as the focal innovation diffuses and system interface needs to increase (see illustration in Fig. 4).

## 5. Conclusions

The paper has shown that nexus building is an important topic for sustainability science and research on low-carbon transitions, shifting the analytical focus from the consequences of nexuses to the processes of creating new connections between systems. Moving beyond abstract typologies of multi-system interactions, the paper developed a more granular microlevel perspective on nexus building, which aimed to capture relevant processes across technological, resource, actor, and institutional dimensions in the system interface. The perspective proposed and the case study confirmed that these multidimensional nexus-building processes are full of conflicts, tensions, misunderstandings, and misalignments in the early deployment phase (beyond subsidized and voluntary experiments in sheltered conditions). In the wider diffusion phase, actors therefore engage in efforts to reduce these conflicts and misalignments, including learning processes, innovation, negotiations, institutional adjustments, or creating new networks and platforms. These efforts and adjustments may be asymmetric between systems (because of differences in power, orientation, and willingness) and incomplete, so that some tensions and problems may continue to exist, forcing actors to innovate and implement suboptimal workaround solutions during the diffusion process.

Our conceptualization and investigation of the between-systems interface domain advances the general understanding of sustainability transitions in several ways. First, it suggests that the importance of system interfaces increases as the focal niche innovation diffuses because it needs more resources from other systems. Second, it shows that locus of tensions during transitions shifts from niche innovations themselves in early phases to the interface between niche innovations and other systems in the diffusion phase (Fig. 4). Third, our case suggests that very rapid diffusion of niche innovations is likely to augment tensions and conflicts in the nexus-building process, especially when the challenges are initially underestimated, which may force actors to deploy suboptimal solutions.

Our framework and insights are relevant and timely because nexus building is likely to become more important in the coming years. The reason is that at least four of the six core pathways for low-carbon transitions identified by the International Energy Agency (1) involve extensive multi-system interactions (50). These include a) electrification, which involves new nexuses between the electricity system and heating (to support heat pumps), mobility (to support electric vehicles), and heavy industry systems (to support electric boilers in chemicals or electric arc furnaces in steel manufacturing); b) alternative fuels and feedstocks (e.g., biofuel, hydrogen, and ammonia) for aviation, shipping, and heavy industry; c) carbon capture and storage for heavy industry, blue hydrogen, and negative emissions; and d) behaviour change including integrated transport systems to support modal shifts and circular economy systems to enhance material efficiency (1, 51). The challenges and processes thus hold wider relevance beyond the specific ferry case.

Our framework and findings are also relevant for policy because policymakers arguably pay too little attention to system interfaces in low-carbon transitions. This is increasingly leading to problems that may delay low-carbon transition pathways, including slow upgrades and extensions in electricity grids that cause increasing waiting lines to connect remote solar and wind parks and new users such as EV charging stations along motorways (52), growing bottlenecks in sourcing critical minerals for EV batteries, solar panels, and electrolyzers (53, 54), and missing connections between the systems involved in production and utilization of hydrogen (55).

To reduce the problems and the risk of delays, policymakers should be more attentive to system interfaces in low-carbon transitions. Based on our framework and findings, we suggest that they should better anticipate the importance of system interfaces and assess potential challenges in a way that considers the properties of the interacting systems (e.g., external pressures, actor strategies, resources, and formal and informal institutions). To avoid problems later on, policymakers should then also analyze and experiment with different ways of building and operating system interfaces to enable early learning processes and stakeholder negotiations. If existing institutions or power asymmetries hamper the involvement of particular actors, policymakers should consider adjustments in regulations or incentive structures. We thus suggest that policymakers should dedicate significant political attention, investments, and efforts to analyzing and addressing interface challenges along our four dimensions early on, instead of waiting for mounting problems during a rushed diffusion process.

While many studies of low-carbon transition pathways understandably focus on focal systems, we hope that our study has highlighted the importance of also analyzing intersystem nexus building and increased the policy awareness of the multidimensional challenges and processes involved. To further conceptualize interface-building processes, future research could fruitfully mobilize work from management studies on tensions in cross-sectoral partnerships (56, 57) and insights from research on institutional work and dynamics of organizational fields (58, 59). Future research should also zoom out to consider multiple interacting system transitions that are happening in parallel. Such coevolving developments in a patchwork of systems are central to achieving net-zero climate targets but only poorly understood (60). Last but not least, future research could focus more on policy mixes and multi-system governance in nexus-building processes.

## Supplementary Material

Appendix 01 (PDF)

## Data Availability

Some study data available (We do not have permission to share our interview data openly. But this data will be made available upon reasonable request from other scholars provided credible provision can be made for protection of the identify of our sources. All other data are quoted in the text or available from cited resources).

## Footnotes

^*^The authors define symbiotic interaction as when both systems benefit from the interaction, such as when the growth of one system leads to growth in the other system due to input–output relations.

^†^While tensions between actors from different systems are discussed in both management studies (56, 57) and institutional theory (58, 59), deep engagement with those theories is beyond the scope of our paper.

## References

[1] IEA, “Net zero by 2050 - A Roadmap for the Global Energy Sector” (International Energy Agency, Paris, 2021).

[2] UNEP, “Emissions gap report 2022: The closing window–Climate crisis calls for rapid transformation of societies” (United Nations Environment Programme, Nairobi Geneva, Switzerland, 2022).

[3] G. Fridgen, R. Keller, M.-F. Körner, M. Schöpf, A holistic view on sector coupling. Energy Policy **147**, 111913 (2020).

[4] M. Robinius , Linking the power and transport sectors—Part 1: The principle of sector coupling. Energies **10**, 956 (2017).

[5] D. Wichelns, The water-energy-food nexus: Is the increasing attention warranted, from either a research or policy perspective? Environ. Sci. Policy **69**, 113–123 (2017).

[6] J. Liu , Nexus approaches to global sustainable development. Nat. Sustainability **1**, 466–476 (2018).

[7] R. Cairns, A. Krzywoszynska, Anatomy of a buzzword: The emergence of ‘the water-energy-food nexus’ in UK natural resource debates. Environ. Sci. Policy **64**, 164–170 (2016).

[8] W. C. Clark, A. G. Harley, Sustainability science: Toward a synthesis. Ann. Rev. Environ. Resources **45**, 331–386 (2020).

[9] T. R. Miller , The future of sustainability science: A solutions-oriented research agenda. Sustainability Sci. **9**, 239–246 (2014).

[10] A. Klitkou, S. Bolwig, T. Hansen, N. Wessberg, The role of lock-in mechanisms in transition processes: The case of energy for road transport. Environ. Innov. Soc. Transitions **16**, 22–37 (2015).

[11] K. C. Seto , Carbon lock-in: Types, causes, and policy implications. Ann. Rev. Environ. Resources **41**, 425–452 (2016).

[12] J. Köhler , An agenda for sustainability transitions research: State of the art and future directions. Environ. Innov. Soc. Transitions **31**, 1–32 (2019).

[13] D. Rosenbloom, Engaging with multi-system interactions in sustainability transitions: A comment on the transitions research agenda. Environ. Innov. Societal Transitions **34**, 336–340 (2020), 10.1016/j.eist.2019.10.003.

[14] K. Konrad, B. Truffer, J.-P. Voß, Multi-regime dynamics in the analysis of sectoral transformation potentials–Evidence from German utility sectors. J. Cleaner Prod. **16**, 1190–1202 (2008).

[15] R. Raven, G. Verbong, Multi-regime interactions in the Dutch energy sector. The case of combined heat and power technologies in the Netherlands 1970–2000. Technol. Anal. Strategic Manage. **19**, 491–507 (2007).

[16] G. Papachristos, A. Sofianos, E. Adamides, System interactions in socio-technical transitions: Extending the multi-level perspective. Environ. Innov. Soc. Transitions **7**, 53–69 (2013).

[17] F. W. Geels, Technological transitions as evolutionary reconfiguration processes: A multi-level perspective and a case-study. Res. Policy **31**, 1257–1274 (2002).

[18] F. W. Geels, Technological Transitions and System Innovations. A Co-Evolutionary and Socio-Technical Analysis (Edward Elgar, 2005), p. 328.

[19] J. Schot, F. W. Geels, Strategic niche management and sustainable innovation journeys: Theory, findings, research agenda, and policy. Technol. Anal. Strategic Manage. **20**, 537–554 (2008).

[20] A. Smith, R. Raven, What is protective space? Reconsidering niches in transitions to sustainability Res. Policy **41**, 1025–1036 (2012).

[21] P. A. David, J. A. Bunn, The economics of gateway technologies and network evolution: Lessons from electricity supply history. Inform. Econ. Policy **3**, 165–202 (1988).

[22] M. L. Tushman, J. P. Murmann, Dominant designs, technology cycles and organizational outcomes. Res. Organ. Behav. **20**, 231–266 (1998).

[23] D. North, Institutions, Institutional Change and Economic Performance (Cambridge University Press, Cambridge, 1990).

[24] M. Smink, S. O. Negro, E. Niesten, M. P. Hekkert, How mismatching institutional logics hinder niche–Regime interaction and how boundary spanners intervene. Technolog. Forecast. Soc. Change **100**, 225–237 (2015).

[25] T. Lawrence, R. Suddaby, “Institutions and institutional work” in The SAGE Handbook of Organization Studies, S. R. Clegg, C. Hardy, T. B. Lawrence, Eds. (SAGE Publications Ltd, London, 2006), pp. 215–254.

[26] L. Fuenfschilling, B. Truffer, The structuration of socio-technical regimes—Conceptual foundations from institutional theory. Res. Policy **43**, 772–791 (2014).

[27] F. W. Geels, J. Schot, Typology of sociotechnical transition pathways. Research Policy **36**, 399–417 (2007).

[28] D. Loorbach, J. Rotmans, The practice of transition management: Examples and lessons from four distinct cases. Futures **42**, 237–246 (2010).

[29] B. van Mierlo, P. J. Beers, Understanding and governing learning in sustainability transitions: A review. Environ. Innov. Soc. Transitions **34**, 255–269 (2020).

[30] D. Lavie, Capability reconfiguration: An analysis of incumbent responses to technological change. Acad. Manage. Rev. **31**, 153–174 (2006).

[31] A. D. Andersen, Towards a new approach to natural resources and development: The role of learning, innovation and linkage dynamics. Int. J. Technol. Learn. Innov. Dev. **5**, 291–324 (2012).

[32] R. Groß , How the European recovery program (ERP) drove France’s petroleum dependency, 1948–1975. Environ. Innov. Soc. Transitions **42**, 268–284 (2022).

[33] J. Summerton, Changing Large Technical Systems (Westview Press, Boulder, San Francisco and Oxford, 1994).

[34] ETC, “Making clean electrification possible: 30 years to electrify the global economy” (Energy Transition Commission, London, England, 2021). Accessed 15 September 2022.

[35] K. M. Eisenhardt, Building theories from case study research. Acad. Manage. Rev. **14**, 532–550 (1989).

[36] Norsk klimastiftelse, “Hvor mange fergesamband er elektriske?” (Norsk klimastiftelse, Bergen, Norway, 2022). https://www.tilnull.no/ferger. Accessed 22 February 2023.

[37] NVE, “Tilknytning med vilkår om utkobling” (The Norwegian Water Resources and Energy Directorate, Oslo, Norway, 2022). https://www.nve.no/reguleringsmyndigheten/regulering/nettvirksomhet/nettilknytning/leveringsplikt/tilknytning-med-vilkaar-om-utkobling/. Accessed 22 February 2023.

[38] Statistics Norway (2019). https://www.ssb.no/en/energi-og-industri/energi/statistikk/elektrisitet. Accessed 6 March 2023.

[39] S. G. Sjøtun, A ferry making waves: A demonstration project ‘doing’ institutional work in a greening maritime industry. Norw. J. Geograp. **73**, 16–28 (2018).

[40] H. Nykamp, A. D. Andersen, F. W. Geels, Low-carbon electrification as a multi-sector transition: A socio-technical analysis of Norwegian maritime transport, construction, and chemical sectors. Environ. Res. Lett. **18**, 094059 (2023).

[41] A. Fenstad, “Has to find collective charging solution for medium-sized vessels (Skal finne felles ladeløsning for mellomstore fartøy)” in Teknisk Ukeblad, J. M. Moberg, Ed. (Teknisk Ukeblad Media AS, Oslo, Norway, 2021).

[42] T. Stensvold, “Battery swapping with robot cuts emissions in hurtigbåter (Batteribytte med robot gjør hurtigbåter utslippsfrie)” in Teknisk Ukeblad, J. M. Moberg, Ed. (Teknisk Ukeblad Media AS, Oslo, Norway, 2021).

[43] A. Fenstad, “Wants to replace ferry chargers with self-driving battery packs (Vil bytte ut fergeladere med selvkjørende batteripakker)” in Teknisk Ukeblad, J. M. Moberg, Ed. (Teknisk Ukeblad Media AS, Oslo, Norway, 2020).

[44] T. Stensvold, “Battery ferries: Charging systems often fail (Batteriferger: Ladesystemer svikter ofte)” in Teknisk Ukeblad, J. M. Moberg, Ed. (Teknisk Ukeblad Media AS, Oslo, Norway, 2020).

[45] O. P. Pedersen, “Butchers revenue model–Does not solve the problems (Slakter inntektsrammeforslag–løser ikke strømproblemene)” in EUROPOWER (NHST Media Group, Oslo, Norway, 2022).

[46] Fornybar Norge, Veileder: Nettilknytning av ferger, busser og hurtigbåter (Fornybar Norge (previously Energi Norge), Oslo, Norway, 2020). https://www.fornybarnorge.no/publikasjoner/veileder/2020/nettilknytning-av-ferger-busser-og-hurtigbater/. Accessed 12 October 2022.

[47] J.-A. Tomasgard, “The solution of the future? Shared energy stations for boat and car (Framtidens løsning? Felles energistasjon for båt og bil)” in Teknisk Ukeblad (Teknisk Ukeblad Media AS, Oslo, Norway, 2021).

[48] L. Kanger, F. W. Geels, B. Sovacool, J. Schot, Technological diffusion as a process of societal embedding: Lessons from historical automobile transitions for future electric mobility. Transp. Res. Part D **71**, 47–66 (2019).

[49] D. Bauknecht, A. D. Andersen, K. T. Dunne, Challenges for electricity network governance in whole system change: Insights from energy transition in Norway. Environ. Innov. Societal Transitions **37**, 318–331 (2020).

[50] A. D. Andersen, F. W. Geels, Multi-system dynamics and the speed of net-zero transitions: Identifying causal processes related to technologies, actors, and institutions. Energy Res. Soc. Sci. **102**, 103178 (2023).

[51] McKinsey, “The net-zero transition: What it would cost, what it could bring” (McKinsey Global Institute, New York, NY, 2022).

[52] A. Mooney, Gridlock: How a lack of power lines will delay the age of renewables. Financial Times (2023). https://www.ft.com/content/a3be0c1a-15df-4970-810a-8b958608ca0f. Accessed 15 June 2023.

[53] IEA, “The role of critical minerals in clean energy transitions” (IEA, Paris, 2021).

[54] H. Gong, A. D. Andersen, The role of natural resources in accelerating net-zero transitions: Insights from EV lithium-ion battery technological innovation system in China, in TIK Working Papers on Innovation Studies (Center for Technology, Innovation, and Culture, University of Oslo, Oslo, Norwary, 2022) p. 1–38.

[55] T. Mäkitie, J. Hanson, M. Steen, T. Hansen, A. D. Andersen, Complementarity formation mechanisms in technology value chains. Res. Policy **51**, 104559 (2022).

[56] D. Dentoni, J. Pinkse, R. Lubberink, Linking sustainable business models to socio-ecological resilience through cross-sector partnerships: A complex adaptive systems view. Business Soc. **60**, 1216–1252 (2021).

[57] M. Kuhlmann, J. Meuer, C. R. Bening, Interorganizational sensemaking of the transition toward a circular value chain. Organ. Environ. **36**, 411–441 (2023). 37655120 10.1177/10860266231162057 PMC10465310

[58] N. Fligstein, D. McAdam, Toward a general theory of strategic action fields. Sociol. Theory **29**, 1–26 (2011).

[59] S. Furnari, Institutional fields as linked arenas: Inter-field resource dependence, institutional work and institutional change. Hum. Relations **69**, 551–580 (2016).

[60] A. D. Andersen , Faster, broader, and deeper! Suggested directions for research on net-zero transitions. Oxford Open Energy **2**, oiad007 (2023).

