# Supplementary material for: Building multi-system nexuses in low-carbon transitions: Conflicts and asymmetric adjustments in Norwegian ferry electrification
Source: Proc Natl Acad Sci U S A. 2023 Nov 13;120(47):e2207746120. doi: 10.1073/pnas.2207746120 (PMC10666111; doi:10.1073/pnas.2207746120)
Supplement: Supplementary file 1 — Appendix 01 (PDF) [file pnas.2207746120.sapp.pdf]

Table A: Overview of interviewees

Code	***Name***	***Type of actor***	***System domain***	***Interviewee role***
**R1**	ABB	Technology supplier/ system integrator	Maritime	Sales Manager
**R2**	BKK	DSO	Electricity	Head of Electrification
**R3**	Energi Norge	Business association for electricity industry	Electricity	Senior Advisor
**R4**	ENOVA	Public green Innovation agency	Both	Senior Advisor
**R5**	Fjord1	Ferry operator	Maritime	Technology and Project Director
**R6**	Greenstat	Green hydrogen company	Electricity	Chief Executive Officer
**R7**	Hordaland county	County owning ferry routes	Maritime	Director, Division of Industry, Planning, and Innovation
**R8**	KS Bedrift & Norske havner	Business association for industry and harbours	Maritime	Energy policy officer
**R9**	Maritime Forum	Advocacy group for maritime industry	Maritime	Chief Executive Officer
**R10**	MøreNett (1)	DSO	Electricity	Senior engineer, Grid development
**R11**	Mørenett (2)	DSO	Electricity	Head of board and CEO of electric utility
**R12**	NCE Maritime	Maritime Cluster organisation	Maritime	Chief Executive Officer
**R13**	Norled	Ferry operator	Maritime	Chief Technology Officer
**R14**	Norwegian Maritime Authority	Regulator maritime transport system	Maritime	Senior Advisor
**R15**	NVE (Norges vassdrags- og energidirektorat)	Regulatory agency electricity system	Electricity	Senior Advisor
**R16**	REN	Business association for DSOs	Electricity	Project manager
**R17**	Skyss	Regional procurer of ferry services	Maritime	Special advisor
**R18**	NPRA (National Public Road Administration)	Main regulator in transport system	Maritime	Senior advisor, procurement of ferry services
**R19**	The Fjords	Ferry operator	Maritime	Chief Executive Officer
**R20**	Torghatten	Ferry operator	Maritime	Chief Executive Officer
**R21**	Trondheim Havn	Harbour company	Maritime	Chief Technology Officer
**R22**	Trønder energinett	DSO	Electricity	Senior advisor, Grid development
**R23**	Wartsila	Global Technology supplier	Maritime	Technical Director
**R24**	Zero Emission Resource Organisation (ZERO)	Environmental NGO	Both	Technology Manager, Project leader zero emission maritime sector
**R25**	Bergen Havn	Port company		Chief Executive Officer of Bergen Port
**R26**	DNV	Consultancy company	Both	Vice President, DNV Maritime
**R27**	Maritime Bergen	Local advocacy group for maritime industry	Maritime	Chief Executive Officer
**R28**	Siemens	Global Technology supplier	Maritime	Head of Sales Offshore and maritime

Table B: Overview of background documents

No.	Document title	Source
**D1**	Best practice connecting buses, ferries and speed boats to the electricity grid (2020). *(Beste praksis for tilknytning av busser, ferger og hurtigbåter)*	Energi Norge
**D2**	Report: Is there capacity in the grid for electric cars, buses and ferries? (2017) *(NVE rapport: Har strømnettet kapasitet til elektriske biler, busser og ferger?)*	The Norwegian Water Resources and Energy Directorate (NVE)
**D3**	Report: Cost of zero- and low-carbon solutions in municipal ferry routes (2020). *(Merkostnader som følge av lav- og nullutslippsløsninger i fylkeskommunale ferjesamband)*	DNV / Ministry of Transport
**D4**	Report: Low- and zero- emission criteria in public procurement of ferry and speed boat services? (2022) *(Lav – og nullutslippskrav ved anskaffelse av ferger og hurtigbåter)*	Agency for Public and Financial Management (DFØ)
**D5**	White paper: Greener and smarter – the maritime industry for tomorrow (2020). *Meld. St. 10 (2020–2021) Grønnere og smartere – morgendagens maritime næring.*	Norwegian Ministry of Trade, Industry and Fisheries
**D6**	The Government's action plan for green shipping (2019). (*Regjeringens handlingsplan for grønn skipsfart)*	Government/ Multiple ministries
**D7**	Electrification of shipping: Status for shore power in gateway ports (2020) (*Elektrifisering av skipsfarten Status for landstrøm i stamnetthavnene*)	Zero Emission Resource Organisation / Grønt skipsfartsprogram
**D8**	Report: Maritime opportunities – blue growth for a green future: The government maritime strategy (2015). (*Maritime muligheter – blå vekst for grønn fremtid Regjeringens maritime strategi*)	Norwegian Ministry of Trade, Industry and Fisheries
**D9**	Maritim 21 - strategy for research, development and innovation in the maritime sector (2022)	Norwegian Ministry of Trade, Industry and Fisheries / Norwegian research council
**D10**	Maritim 21 An integrated maritime strategy for research, development and innovation (2016) *(Maritim 21: En helhetlig maritim strategi for forskning, utvikling og innovasjon)*	Norwegian Ministry of Trade, Industry and Fisheries/ Norwegian research council
**D11**	Maritim 21: An integrated maritime research and innovation strategy (2010) *(Maritim 21: En Helhetlig Maritim Forsknings- og Innovasjonssatsing)*	Norwegian Ministry of Trade, Industry and Fisheries/ Norwegian research council
**D12**	Infrastructure for electric transportation: What responsibilities should grid companies have? (2021) *(Infrastruktur for elektrisk transport: Hvilket ansvar skal nettselskapene ha?)*	AFRY Management Consulting: Oslo.

Table C: Overview of industry meetings

No.	Event title & description	Organizer
**1**	Zero-emission maritime sector (04/05-2017, Oslo) (*Seminar: Utslippsfri maritim sektor*), https://zero.no/seminar-utslippsfri-maritim-sektor/	Zero Emission Resource Organisation (ZERO)
**2**	The electrification of the maritime sector (15/10-2018, Bergen). (*Seminar: Elektrifiseringen av maritim sektor*), https://zero.no/seminar-elektrifiseringen-av-maritim-sektor/	Zero Emission Resource Organisation (ZERO)
